# Innovation through neurodiversity: Diversity is beneficial

**DOI:** 10.1177/13623613231158685

**Published:** 2023-03-07

**Authors:** Harriet Axbey, Nadin Beckmann, Sue Fletcher-Watson, Alisdair Tullo, Catherine J Crompton

**Affiliations:** 1 Durham University, UK; 2 University of Edinburgh, UK

**Keywords:** autism, creativity, diffusion chains, neurodiversity, innovation

## Abstract

Those experiencing high rapport or strong social connection are more likely to copy each other, or emulate each other’s ideas, either consciously or sub-consciously. In this study, we use this phenomenon to examine whether neurotype match or mismatch impacts degree of imitation in a creative task. We asked 71 participants in neurodiverse pairs (including both autistic and non-autistic participants) and single-neurotype pairs (both autistic or both non-autistic), where one participant builds and one observes, to build the tallest possible tower from dried spaghetti and plasticine. We measured the height of each tower and photographed them to create a stimulus set. We then asked independent raters (*n* = 351, 62 autistic) to rate towers for degree of similarity. We hypothesised that lower similarity scores would be generated for towers created by people in neurodiverse pairs, showing positive innovation. Results showed towers built in the neurodiverse condition had least similarity, whereas towers built in the autistic and non-autistic conditions were significantly more similar. There was no difference in performance (height of tower) based on condition. Our results are the first to examine creativity within single-neurotype and neurodiverse pairs; they indicate that neurological diversity may be beneficial within a group setting. Subsequent research is required to examine how this interacts with divergent communication styles.

**Lay abstract:**

Neurodivergences such as autism have been previously viewed from a negative, ‘deficit’, perspective. However, research is beginning to show the benefits of being autistic, and the positive outcomes of neurodiverse interactions. Diversity in the way we think can lead to diversity in the outcomes we produce. In this study, we asked independent raters to compare the similarity of towers built by autistic and non-autistic individuals in single-neurotype (both people were autistic or both people were non-autistic) and neurodiverse (one autistic person and one non-autistic person) pairs, to see whether people would be more or less likely to copy someone who shared their diagnostic status. Our results showed there was the least similarity in design in the neurodiverse pairs; people were less likely to copy the design of the previous builder if that person had a different autistic status to themselves. This could imply people felt more confident in copying someone with a similar neurotype, mirroring results from rapport studies where autistic individuals reported greater rapport with other autistic participants than with non-autistic participants. This also shows there was more evidence of creativity in designs, and innovation from stimulus design (the tower they had watched being built) when the pairs had different autistic diagnoses. This could inform practice and support involving autistic people, encouraging education and care providers to create more diverse methods and designs for support mechanisms, content delivery, and research data collection.

## Introduction

Autism is an example of neurodivergence; where there are individuals with different neurotypes, such as in a group of people, some of whom are autistic and some of whom are not, this is called neurodiversity. Most research examines autistic social behaviour and cognition at an individual level, through direct comparison of autistic and non-autistic people (Gernsbacher & Yergeau, 2019). However, in doing so, this neglects the role of interactive and interpersonal dynamics, which are an essential part of understanding neurodiversity. There is evidence that stronger social connections can lead to imitation and emulation.

The ability to both replicate from others and innovate has helped human survival to the present day, and is necessary in our development both physically and socially (Hopper et al., 2010; Horner et al., 2006). Innovation has been studied experimentally using the diffusion chain method, a method of studying the cultural transmission of information across generations, in a way similar to the children’s game of ‘telephone’ (Carr et al., 2015). This method involves pairs of participants completing a task together within a larger ‘chain’ of participants (Crompton, Ropar, et al., 2020). This method facilitates examination of the evolution of ideas, and how they develop through ‘generations’ of participants (Caldwell & Millen, 2008a).

In this study, we aimed to investigate social connectivity and similarity through observation, to see whether there would be greater imitation within single-neurotype pairs compared with mismatched pairs of autistic and non-autistic people. Participants were asked to rate the similarity of Spaghetti Towers (Caldwell & Millen, 2008a, 2010) that had been created by autistic and non-autistic individuals, during a diffusion chain procedure. The towers were created by people who had previously watched another individual make a tower, while being observed by the next participant in the chain. In one condition, the observer and first tower-maker were both autistic, in another, they were both non-autistic, and in a third, the pair were mismatched (autistic and non-autistic). We also compared the heights of the towers created by participants in each of the three groups as a performance indicator.

We hypothesised that participants who were in mismatched pairs would create less similar tower designs as indicated by lower similarity scores given by raters. If their tower was less similar, this could suggest they had innovated from their stimulus tower; innovation in this context is considered positive and beneficial, as it shows diversity and creativity in outcomes. We also examined tower height, but had no a priori hypothesis about this.

## Method

### Creating the photo stimuli

Seventy-one photo stimuli were created for use in this study during a research day as part of a wider project looking at diverse social intelligence (Crompton, Ropar, et al., 2020). Each photo featured a tower built from dried spaghetti and plasticine that had been created by a participant.

The stimuli were created during a diffusion chain study (Crompton, Ropar et al., 2020; Flynn & Whiten, 2008). Chains consisted of eight participants (seven in one case due to attrition) and were in one of three conditions: autistic, non-autistic, and neurodiverse (alternating autistic and non-autistic participants, beginning with a non-autistic participant). Participants in each chain were asked to build a tower as tall as possible within the space of five min: they took turns both observing and building a tower. For example, Participant Three in the chain would watch Participant Two build a tower out of spaghetti and playdough. Then, Participant Two would leave the room and their tower would be deconstructed. Participant Four would enter the room and watch Participant Three building a tower. This pattern continued through the entire chain.

**Figure 1.**
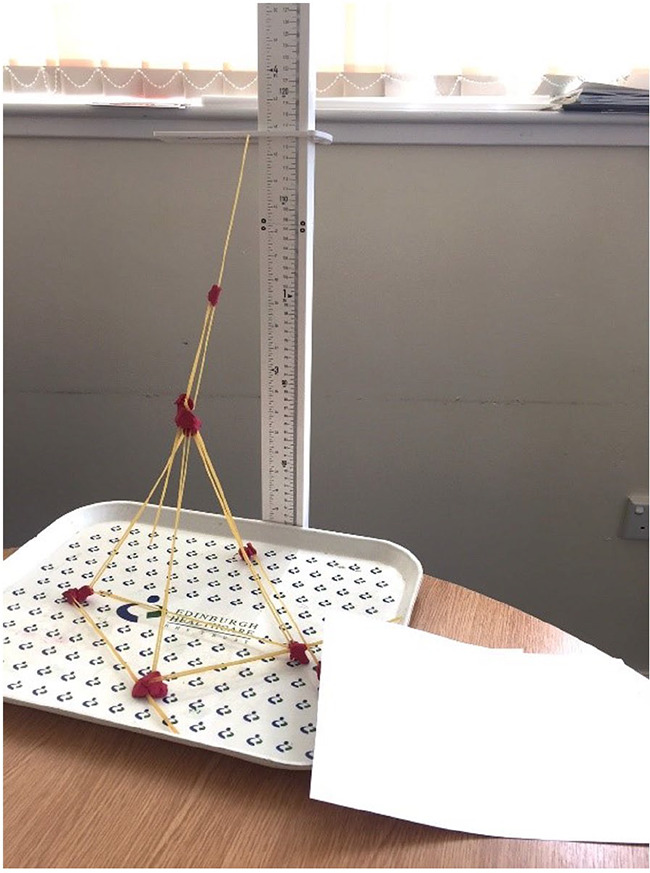
An example photo stimulus, indicating a spaghetti tower created by a participant.

Before being deconstructed, towers were measured in centimetres by a researcher, using a metre stick. This task was completed by 71 adult participants (35 autistic) at the University of Edinburgh. An example of one of the towers is shown in Figure 1.

### Ratings of similarity

We used an experimental design using independent raters to judge the similarity of task outcomes (towers built) by autistic and non-autistic individuals from the observation and building task. The experimental factor was the condition under which the original stimulus tower was built. This was a computer-based task, administered online. Full code for the task programme and the stimuli used can be found in Tullo et al. (2022).

**Figure 2.**
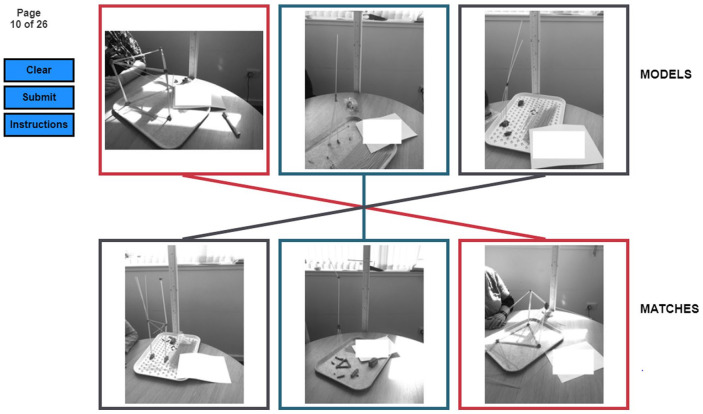
A screenshot from the task.

The task presented raters with six images at once (see Figure 2). Each block of six images contained two consecutive images from three different types of diffusion chains (autistic, non-autistic, and neurodiverse). Participants were not informed whether the stimuli were created by autistic or non-autistic participants, nor did they know what conditions (i.e. matched or mismatched neurotype observer) applied when the tower was built. Raters were asked to match images into pairs based on their similarity, instructions read as follows:

In a previous study we asked people to build towers out of spaghetti and playdough. Now, we want to ask you to decide how similar the towers were to each other. We’re going to show you six photos of spaghetti towers; three on the top row and three on the bottom row. Your job is to pair up the pictures, according to how similar you think they are. Click on a picture in the top row, and then click the tower on the bottom row that you think is most similar, to create a linked pair. You must make three links to move on to the next screen. Sometimes there will be one photo that really doesn’t look like any of the other towers on the page. You still have to pick a pair for it! If you’re not happy with your choices click ‘Clear’ to remove them and start again. Once you click ‘Submit’ your choice is recorded and you can’t go back. Please don’t use your browser’s ‘back’ button as this will exit the experiment!

A correct match was scored as 1, and an incorrect match scored as 0. A correct match was defined as the rater matching two pictures from the same diffusion chain. Therefore, the mean average score for each diffusion chain (autistic, non-autistic, neurodiverse) is found between 0 and 1.

Raters for the similarity judgements were recruited via Prolific and Twitter. Of the 351 raters, 62 reported being autistic (43 diagnosed, 19 self-diagnosed). Participants’ ages ranged from 18 to 71 years (mean (M) = 32, standard deviation (SD) = 12.14), with 215 male, 127 female and 9 identifying otherwise. Specific data on race/ethnicity and socioeconomic status were not recorded. Raters recruited via Prolific were reimbursed for their time (£0.84 for 8 min), and participants recruited via Twitter had the opportunity to be entered into a draw for a £50 gift voucher.

## Ethical approval

Ethical approval for the diffusion chain stimuli creation was granted by the University of Edinburgh, and approval for collecting the similarity judgements from independent raters was approved by Durham University.

## Results

**Figure 3.**
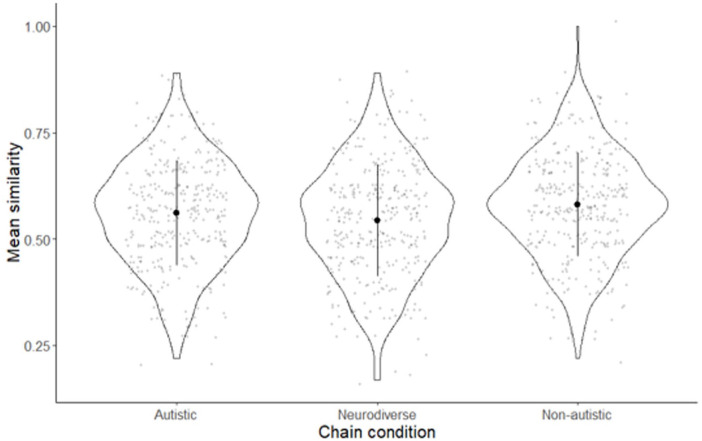
Mean similarity of towers as judged by 351 raters.

The mean similarity rating (mean average score for each diffusion chain) was the highest for towers built in the non-autistic condition (M = 0.580, SD = 0.121) followed by the autistic condition (M = 0.560, SD = 0.123) and finally, the neurodiverse condition (M = 0.544, SD = 0.130) (Figure 3).

Perceived similarity of towers differed significantly between conditions, as shown by a repeated measures analysis of variance (ANOVA) (*F*(696) = 5.968, *p* < 0.05; $\eta_{p}^{2}$ = 0.17), using age and gender as covariates. A post hoc analysis with the Bonferroni adjustment determined that the similarity between towers in the neurodiverse (ND) condition was significantly less than that in the autistic (A) condition (mean difference ND minus A = −0.016 (95% confidence interval (CI) = −0.031 to −0.001), *p* < 0.05) and the non-autistic (NA) condition (mean difference ND minus NA = −0.036 (95% CI = −0.051 to −0.021), *p* < 0.001). The similarity between the non-autistic and the autistic conditions also differed significantly (mean difference A minus NA = −0.020 (95% CI = −0.036 to −0.004), *p* < 0.05).

A Cohen’s *d* calculation using pooled standard deviations showed that the difference between the similarity judged between the autistic and neurodiverse conditions’ chains had an effect size of *d* = −0.126 (SD_pooled_ = 0.127). The effect size between the non-autistic and the neurodiverse conditions was larger, at *d* = −0.284 (SD_pooled_ = 0.126).

The average tower height across conditions was 57.68 cm (SD = 22.13 cm). Descriptively, the mean height of towers was the highest in the non-autistic condition (61.38 cm, SD = 18.69 cm) and the lowest in the neurodiverse condition (53.08 cm, SD = 22.45 cm); towers in the autistic condition averaged 58.61 cm (SD = 25.23). A one-way ANOVA found there was no significant difference between the three conditions (*F*(40, 30) = 1.23, *p* = 0.28).

## Discussion

The results show that there was a small but significant difference in perceived similarity between towers in the neurodiverse condition and towers in the single-neurotype conditions, with non-autistic towers being the most similar of the three conditions. We therefore tentatively accept our hypothesis that similarity would be the lowest in the neurodiverse groups.

In this study, a greater range in design (as indicated by lower similarity between towers) is a better outcome as it shows greater creativity. Our results indicate that neurodiversity creates more diverse solutions, adding to the ‘value in diversity’ model that suggests that diverse groups will produce better outcomes (Herring, 2009; Hofstra et al., 2020).

Furthermore, the greater similarity rated within the single-neurotype conditions suggest that participants were more likely to imitate from those who shared a similar neurotype. This could be related to greater rapport between participants, if they chose to replicate the design of the person they were observing, especially if they identified with the identity of the builder, given that their autistic status was known (Brewer, 1979; Matthews et al., 2012). Using the same sample set, greater rapport between those with similar neurotypes was reported during a separate task, so this could be transferred across to this tower-building task (Crompton, Sharp, et al., 2020).

Imitation and emulation are not always beneficial, as we need creativity and innovation in many areas, such as business. Diversity within a group (including *neuro*diversity) and a diverse workforce can add productivity, creativity and even profitability, as research shows that such diverse workforces lead to increased sales, more customers, and greater relative profits (Bigozzi et al., 2016; Herring, 2009). We argue that these findings suggest neurodiversity produces more innovative and diverse designs as an output to a tower-building task. The diffusion chain method for the collection of the stimulus data helped to replicate the natural transmission of cultural and design ideas, and is useful as it shows cumulative effects (Caldwell & Millen, 2008b; Flynn, 2008). Within research, those from under-represented groups are shown to produce higher rates of scientific novelty, and yet these novel contributions are often not recognised, or given due credit (Hofstra et al., 2020). As a minority group, autistic individuals’ contributions may be overlooked or undervalued, which should be considered when looking at observation and similarity-based tasks such as these. While we know that individually, neurodivergent people may be more creative in how they complete tasks (Bigozzi et al., 2016), we do not know whether neurodiversity within groups leads to improved innovation and creative problem-solving. This could be explored further in future research.

There were no significant differences between the heights of the towers built across conditions. This shows that no condition achieved ‘worse’ than another, that is, task performance did not vary significantly based on neurotype. This sheds new light on theories that present autism and neurodivergences as ‘deficit’; theories which are empirically questionable, yet still pervade to create harmful societal impacts (Gernsbacher & Yergeau, 2019).

This study does have limitations, which should be addressed by future research. First, this study only used one stimulus set. The study could be repeated with an additional stimulus set to examine creativity across different types of tasks. Second, although all towers were built in the same room, on the same table, and therefore the images had the same background and lighting, the images were presented to participants in black and white. This was necessary as in the stimulus photos, towers had been created with different colours of plasticine, which may have meant raters based their similarity pairings on colour, rather than structure. Future studies could repeat this method using a full colour stimulus set. Finally, as this is inherently an observation-based rather than collaborative task, it would be interesting to study the effects of being watched while building the towers. Although the first participant in each chain did not watch anyone, and the final participant did not have an observer, no participant completed the task completely independent of another. Further studies could include a control condition, where the builders construct their towers alone, without having observed another and without an observer.

Previous studies on replication and innovation have shown innovation to be rare, especially as age increases (Carr et al., 2015). Therefore, the significant effects of neurodiversity on creative outcomes here show some exciting prospects for the field of innovation studies. Our results are the first to examine innovation and creative thinking within single-neurotype versus neurodiverse pairings, and indicate that neurological diversity may be beneficial in this way. Subsequent research is required to examine how this interacts with divergent communication styles of autistic and non-autistic people.

### Community involvement statement

The lead author on this article is autistic, and the collection of data involved support from the autistic community in Edinburgh.
